# Unleashing the power of complement activation: unraveling renal damage in human anti-glomerular basement membrane disease

**DOI:** 10.3389/fimmu.2023.1229806

**Published:** 2023-09-15

**Authors:** Anqi Tang, Xin Zhao, Tian Tao, Dengpiao Xie, Bojun Xu, Youqun Huang, Mingquan Li

**Affiliations:** ^1^ Clinical Medical College, Chengdu University of Traditional Chinese Medicine, Chengdu, China; ^2^ Department of Nephrology, Hospital of Chengdu University of Traditional Chinese Medicine, Chengdu, China

**Keywords:** anti-glomerular basement membrane disease, complement, autoimmune nephropathy, complement activation, complement therapeutics, emerging therapies

## Abstract

Anti-glomerular basement membrane (GBM) disease is a rare but life-threatening autoimmune disorder characterized by rapidly progressive glomerulonephritis with or without pulmonary hemorrhage. Renal biopsies of anti-GBM patients predominantly show linear deposition of IgG and complement component 3 (C3), indicating a close association between antigen-antibody reactions and subsequent complement activation in the pathogenesis of the disease. All three major pathways of complement activation, including the classical, lectin, and alternative pathways, are involved in human anti-GBM disease. Several complement factors, such as C3, C5b-9, and factor B, show a positive correlation with the severity of the renal injury and act as risk factors for renal outcomes. Furthermore, compared to patients with single positivity for anti-GBM antibodies, individuals who are double-seropositive for anti-neutrophil cytoplasmic antibody (ANCA) and anti-GBM antibodies exhibit a unique clinical phenotype that lies between ANCA-associated vasculitis (AAV) and anti-GBM disease. Complement activation may serve as a potential “bridge” for triggering both AAV and anti-GBM conditions. The aim of this article is to provide a comprehensive review of the latest clinical evidence regarding the role of complement activation in anti-GBM disease. Furthermore, potential therapeutic strategies targeting complement components and associated precautions are discussed, to establish a theoretical basis for complement-targeted therapies.

## 1 Introduction

Over 100 years ago, it was discovered that complement serves as a mediator to activate the innate immune pathway, playing a crucial role in defending against microbial invasion and immune surveillance. However, upon exposure to pathogens or contact with foreign surfaces, improper activation, and immune dysregulation can cause the complement system to shift from a defense mechanism to an attacking system, thereby becoming a primary pathogenic mechanism for numerous diseases (1). Specifically, autoimmune system dysregulation leads to the accumulation of immune complexes that cannot be cleared from the body. Subsequently, the complement system remains continuously activated due to stimulation by immune complexes, recruiting immune cells to launch attacks against self-tissues, resulting in tissue and organ damage and the occurrence of autoimmune diseases (2).

Anti-glomerular basement membrane (anti-GBM) disease is a rare autoimmune disease characterized by the deposition of anti-GBM antibodies in the kidneys and/or lungs, primarily affecting the glomerular capillaries or pulmonary capillaries. The annual incidence of the disease is 1.64 per million, with a 5-year renal survival rate of 34% (3, 4). It is worth noting that low levels of anti-GBM antibodies can also be detected in the general population, even when they fall below the “clinically positive” range (5).

In addition to anti-GBM disease, autoimmune disorders such as rheumatoid arthritis and systemic lupus erythematosus have also exhibited immune responses targeting self-antigens several years before the clinical onset (6, 7). Undeniably, antibodies have the potential to induce immune system attacks and directly damage target organs. However, in cases where antibodies persist for a prolonged period before clinical symptoms appear, complement system dysregulation may serve as a major contributing factor in the amplification of pathogenic factors, ultimately disrupting immune tolerance and leading to disease progression (5, 8). It is worth noting that the presence of antineutrophil cytoplasmic antibody (ANCA) positivity in patients with anti-GBM disease may be associated with more severe clinical manifestations and a poorer prognosis (9, 10). The coexistence of these two conditions could be attributed to intermolecular epitope spreading or the emergence of new antigenic epitopes (11, 12). However, the exact relationship between this phenomenon and complement system activation is currently unclear. Given that the kidney is highly sensitive to complement-mediated damage, it becomes particularly important to clarify the role of complement in anti-GBM disease. Furthermore, targeting the complement system to protect cells or tissues from immune system attack may provide benefits that outweigh the potential harms associated with suppressing complement and weakening the body’s defenses (13). Therefore, targeted therapies aimed at the complement system may hold tremendous potential in the treatment of autoimmune kidney diseases.

## 2 Overview of anti-GBM disease

**Figure 1:**
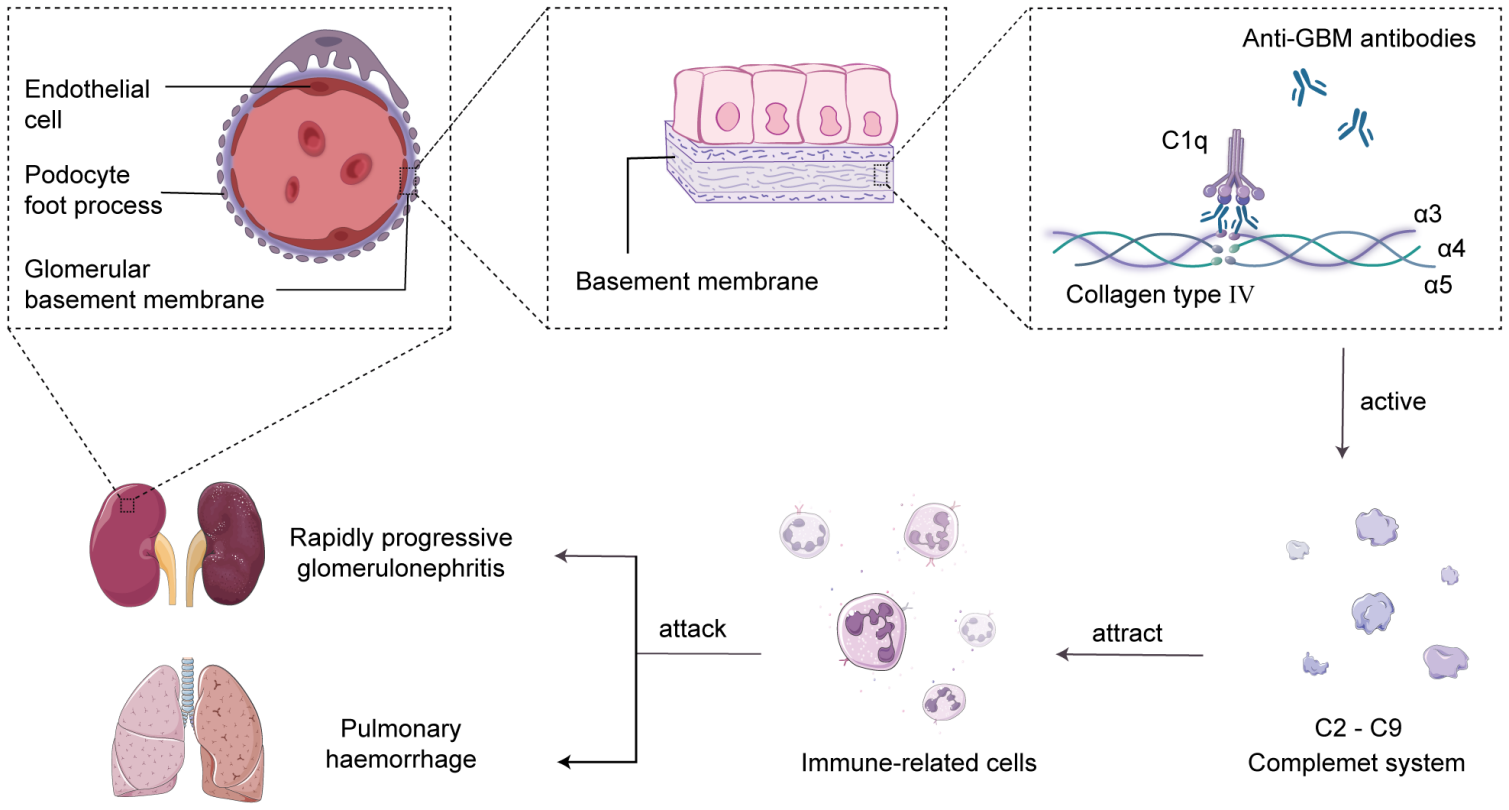
Complement-mediated acute glomerulonephritis and pulmonary hemorrhage. In the human glomerular basement membrane (GBM), the non-collagenous domain of the α3 chain of type IV collagen binds to self-anti-GBM antibodies, forming immune complexes and activating the complement system in situ. This triggers the recruitment of immune cells to attack the GBM and alveolar basement membrane, leading to the development of acute glomerulonephritis and pulmonary hemorrhage syndrome.

The main characteristic of anti-GBM disease is the production of pathogenic autoantibodies called anti-GBM antibodies, which primarily target the type IV collagen antigen in the glomerular and alveolar basement membranes (14). Research has revealed that type IV collagen is a triple-helical structure composed of α3, α4, and α5 chains, and the interactions between non-collagenous domains, such as hydrogen bonds, ionic bonds, and van der Waals forces, lead to the formation of hexameric structures in the GBM (15). The hexameric structure of the GBM serves as a barrier to prevent the interaction between antigens and antibodies. However, under the influence of specific factors, the hexameric structure becomes disrupted, resulting in the exposure of the cryptic antigenic α3 chain non-collagenous domain (α3NC1). This exposed domain is recognized by anti-GBM antibodies, leading to the formation of immune complexes and serving as the initial trigger for the autoimmune response (16). Subsequently, the complement system is activated, leading to a cascade reaction, generating complement cleavage products and inflammatory mediators, recruiting neutrophils to the damaged glomerular basement membrane area, and releasing cytokines and chemokines to attract and activate other immune cells, amplifying the inflammatory response (17, 18). Eventually, the excessive inflammatory reaction causes damage to the glomerular basement membrane and alveolar basement membrane, resulting in acute glomerulonephritis and pulmonary hemorrhage syndrome (19, 20) (**Figure 1**).

It is commonly postulated that a complex interplay of genetic factors, environmental influences, infections, and medications may contribute to the onset of anti-GBM disease (21). It has been observed that α3(IV)NC1 is predominantly localized to the basement membranes of the glomeruli, alveoli, brain, eyes, and inner ear, exhibiting a relatively restricted distribution (22). According to available data, around 90% of patients exhibit renal involvement, primarily presenting as acute glomerulonephritis, and in severe cases, it can progress to renal failure (23). About 40%-60% of patients experience pulmonary inflammation and injury, with pulmonary hemorrhage being the initial symptom in severe cases. Failure to intervene promptly can result in respiratory distress and pose a life-threatening risk (23–25). It is reassuring to note that this percentage has been trending downward in recent studies (15%-35%) due to early detection and early intervention of the disease (3, 4, 26). The treatment of anti-GBM disease, along with other autoimmune disorders in this category, remains a significant challenge. Although treatment modalities such as high-dose corticosteroids, plasma exchange, and immunosuppressive agents can mitigate the progression of anti-GBM disease, they do not offer a definitive cure (14). Of greater concern, our understanding of the disease’s recurrence patterns and triggers remains limited. Anti-GBM disease may relapse at various time points or be influenced by different triggering factors, ultimately leading to the occurrence of end-stage renal failure.

## 3 Complement activation found in anti-GBM disease

Complement, a group of endogenously produced proteins, constitutes an essential component of the immune system, closely intertwined with both innate and adaptive immune responses. The primary functions of the complement system encompass pathogen elimination, inflammatory reactions, antibody-mediated cytotoxicity, and immune modulation (27). Particularly in autoimmune diseases, including anti-GBM disease, the excessive activation of the complement system plays a pivotal role in the onset and progression of the disease. Research suggests that in a mouse model of anti-GBM-mediated glomerulonephritis, mice lacking the Fc-γ receptors chain, which is essential for complement receptor binding, in either inflammatory cells or resident kidney cells, exhibit near-complete complement system paralysis (28). Consequently, no urinary albumin was observed following kidney injury. As anticipated, the absence of albuminuria was also observed in FcR-γ chain and component 3 (C3) double-knockout mice, providing robust evidence for the involvement of the complement system in the pathogenesis of anti-GBM disease (28). Complement primarily induces localized rather than systemic immune responses leading to renal damage (29). Additionally, the levels of serum C3 deposition in glomeruli are positively correlated with the risk of renal failure (30). Complement appears to be linked not only to the extent of renal injury but also to the formation of crescents. For example, glomeruli with crescents demonstrate more intense complement deposition and greater severity of renal damage compared to glomeruli without crescents (31). Specifically, complement activation triggers the formation of the membrane attack complex (MAC), which subsequently mediates endothelial cell apoptosis and paves the way for crucial damage caused by neutrophils and other immune cells to the GBM (32, 33). Ultimately, crescents consisting of endothelial cells, immune cells, and cellular debris are formed within the glomeruli, exacerbating glomerular injury and deterioration of renal function (34–36). Conversely, in mice deficient in C6, the inability to assemble the C6-C9 complex and form the MAC helps them resist renal damage induced by anti-GBM antibodies. This study’s findings offer further confirmation, from an alternative standpoint, of the complement system’s role in renal injury in anti-GBM disease (37). Next, we will discuss the role of complement in anti-GBM disease by examining each of the three complement pathways individually. And evidence for complement activation in anti-GBM patients is presented in **Table 1**.

**Table 1:** Evidence for complement activation in anti-GBM disease.

Evidence	References	Key findings
Complement involved in the pathogenesis of anti-GBM	(28)	In a mouse model of anti-GBM-mediated glomerulonephritis, FcR-gamma-chain (-/-) mice were unable to bind to complement receptors, which in turn failed to activate the complement system and therefore failed to produce albuminuria.
	(29)	Patients with anti-GBM diseases have elevated plasma and urine levels of the terminal complement complex C5b-9.
	(30)	Levels of C3 deposition in glomeruli positively correlate with risk of renal failure.
	(31)	Higher incidence of complement component deposition in crescent-shaped glomeruli.
	(37)	C6-deficient mice are unable to form membrane attack complex, which attenuates anti-GBM antibody-induced kidney injury.
Activation of the Classical pathway	(28)	C1q knockout mice have significantly reduced kidney injury.
	(38)	C1q and anti-C1q antibodies bind to form immune complexes and are deposited in the glomeruli, leading to increased renal injury in mice pretreated with anti-GBM antibodies.
	(39)	C1q was detected in the glomeruli of almost all anti-GBM patients examined, where it was deposited linearly along the glomerular capillary wall and Bowman’s capsule and co-localized with the terminal complement complex C5b-9.
Activation of the lectin pathway	(29)	MBL levels are elevated in plasma and urine in anti-GBM patients.
	(39, 40)	Renal biopsy shows diffuse deposition of MBL in glomeruli.
	(41)	Renal tissue injury is attenuated in both MASP-2-deficient and MASP-2 inhibitor-treated mice
	(42, 43)	Circulating anti-GBM antibodies (mainly IgG4 subclass) activate the lectin complement pathway
Activation of the alternative pathway	(28, 44)	When the classical and lectin pathways are blocked, complement can be activated by alternative pathways.
	(45)	Factor B is deposited linearly and granularly along the glomerular capillary wall and is highly co-localized with C5b-9.
		In crescentic glomeruli, factor B is deposited at a higher rate.

anti-GBM, Anti-glomerular basement membrane; MBL, Mannose-binding lectin; MASP-2, MBL-associated serine proteases 2.

## 4 Complement activation pathways in anti-GBM disease

### 4.1 The classical pathway

**Figure 2:**
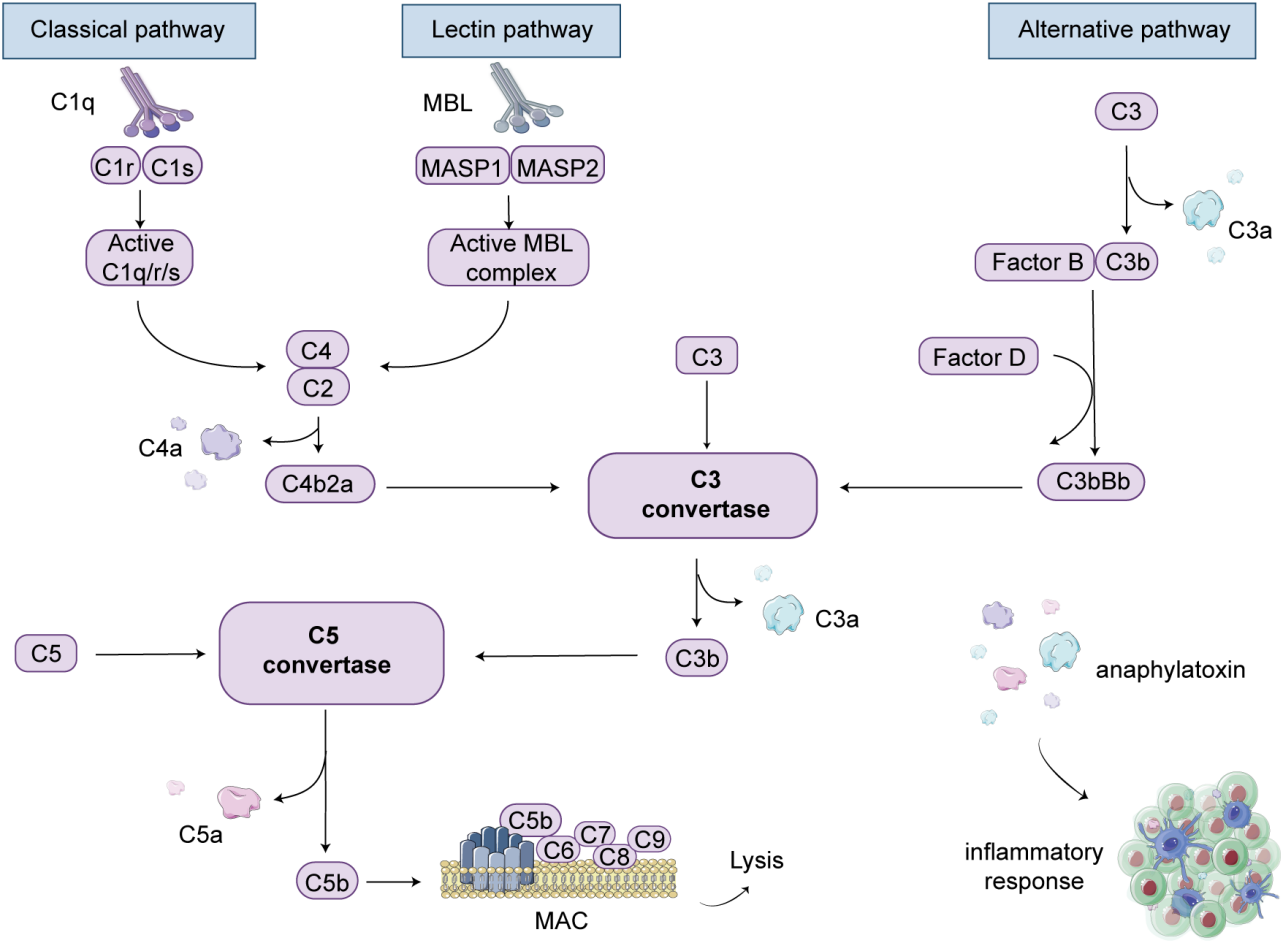
Complement cascade schematic illustration. There are three pathways involved in complement-mediated renal involvement, namely the classical, lectin, and alternative pathways. The classical pathway can be activated by the complex formation of antigens and antibodies. When mannose-binding lectin (MBL) binds to serine proteases, known as mannose-associated serine proteases (MASP1 and MASP2), the lectin pathway is activated. The alternative pathway is initiated when complement C3 covalently binds to microbial surfaces and undergoes cleavage, resulting in the formation of C3b. Each pathway ultimately generates active C3 convertases, leading to the cleavage of C3 into C3a and C3b fragments. C3b can then interact with C4b2a or C3bBb, generating C5 convertases. Under the action of C5 convertases, C5 is cleaved into C5a and C5b. C5b binds to cell membranes and, along with C6, C7, C8, and C9, forms the membrane attack complex (MAC), leading to cell lysis. Additionally, complement products such as C3a, C4a, and C5a act as anaphylatoxins and chemotaxis, attracting and activating immune cells, thereby inducing an inflammatory response.

The complement system is primarily activated through three main pathways: the classical pathway, the lectin pathway, and the alternative pathway. In patients with anti-GBM disease, IgG antibodies are produced, which subsequently target self-antigens, forming immune complexes that bind with C1q. This sequential activation involves complement components such as C4 and C2, leading to the formation of C3 convertase and C5 convertase. Ultimately, the terminal pathway of the three pathways is activated, resulting in the cleavage of C5 into C5b, which then associates with C6, C7, C8, and C9 to form the MAC (46). The MAC formation creates pores on the cell surface, inducing cell lysis and subsequent death of cells or pathogens. This process is regarded as the activation of the classical complement pathway (**Figure 2**).

Research has demonstrated that in the presence of C1q, administration of anti-C1q antibodies results in the binding and deposition of C1q and anti-C1q antibodies in the glomeruli, leading to increased renal damage in anti-GBM antibody-pre-treated mice (38). Moreover, in patients with the anti-GBM disease, C1q deposition along the glomerular capillary wall (GCW) and Bowman’s capsule is detectable in nearly all examined glomeruli, and it co-localizes well with the terminal complement complex C5b-9 (39). In contrast, in a model of renal injury induced by exogenous anti-GBM antibodies, mice deficient in C1q exhibit significantly reduced renal damage (28). This outcome can be partially attributed to the blockade of classical pathway initiation following C1q knockout, thereby inhibiting complement activation and preventing renal injury. However, conventional renal biopsy examinations often fail to demonstrate C1q deposition in immunofluorescence tests (47). Additionally, other research has not found a correlation between the intensity of C1q deposition and the clinical characteristics of patients (39). One potential explanation is that, compared to other autoimmune diseases such as systemic lupus erythematosus, anti-C1q antibodies in anti-GBM patients are generally present at lower levels, making it difficult for them to bind to C1q and deposit in the kidneys (38).

### 4.2 The lectin pathway

Mannose-binding lectin (MBL) is an important soluble pattern recognition molecule that activates the lectin pathway. In contrast to the classical pathway, which primarily relies on antigen-antibody interactions, the lectin pathway primarily depends on the binding of lectins on the pathogen surface to activated lectin receptors on the cell surface, and this binding typically occurs on immune cell surfaces (48). Specifically, when the MBL complex binds to the surface of pathogens, MBL-associated serine proteases (MASP-1 and MASP-2) are activated, initiating the complement cascade, leading to the cleavage and activation of C4 and C2, and subsequently activating the complement system (49) (**Figure 2**). The lectin pathway primarily contributes to the inflammatory response by activating immune cells and releasing inflammatory mediators to combat pathogens (50). In contrast, the classical pathway not only triggers an inflammatory response but also directly destroys pathogens and marks them for clearance by immune cells. Overall, these two pathways play complementary roles in the immune system.

In anti-GBM patients, the elevation of plasma and urinary MBL levels indicates the involvement of the lectin pathway (29). It has been reported that MBL diffusely deposits in the glomerular capillary walls, mesangial areas, GBM, and even crescents (39, 40). MBL co-localizes with partial C4d but not with C5b-9. Based on this, a study suggests that the lectin pathway may not be involved in complement activation in human anti-GBM disease (45). However, considering that MBL primarily participates in the early stages of complement activation, its triggering role in complement activation cannot be ruled out, even though it does not co-localize with C5b-9. Indeed, both MASP-2 deficient mice and mice treated with MASP-2 inhibitors exhibit reduced tubulointerstitial damage and decreased proteinuria, demonstrating the protective effect of inhibiting the lectin pathway on the kidneys (41). Previously, anti-GBM antibodies were thought to mainly belong to the immunoglobulin G subclass 1 (IgG1). Interestingly, there have been reports suggesting the presence of circulating anti-GBM antibodies predominantly of the IgG4 subclass, which may lead to false-negative results in immune detection of anti-GBM disease (51, 52). Despite IgG4 subclass antibodies being considered to have lower complement activation capacity due to their inability to bind to C1q, they can still activate the lectin complement pathway by binding to MBL, leading to complement system activation and substantial deposition in the kidneys (42, 43).

### 4.3 The alternative pathway

Compared to the other two pathways, the alternative pathway does not require the involvement of antibodies or lectins. Instead, it primarily relies on the activation or cleavage of endogenous molecules within the body, such as complement C3, factor B, factor D, and others (53). This also means that the activation of the alternative pathway is largely a spontaneous and non-specific process. Specifically, C3 in the plasma is cleaved into C3a and C3b. C3b, along with blood proteins like factor B and factor D, binds to specific surface structures of microbes and self-molecules in the bodily fluid, forming the C3 convertase and activating the terminal complement pathway (**Figure 2**). It is important to note that the alternative pathway and the classical pathway are not isolated entities; they can mutually influence and cross-activate each other under certain circumstances (54). For example, specific surface structures of pathogens may simultaneously activate these two pathways, thereby triggering a more robust immune response (55).

In the anti-GBM model, the occurrence of inflammatory cell infiltration and renal damage has been observed in mice with a specific knockout of the shared complement component C4 in the classical and lectin pathways. Similarly, in mice with a double knockout of C1q and C4, deposition of C3 complexes can also be detected (28, 44). These facts demonstrate that despite the blockade of the classical and lectin pathways, complement activation can still occur solely through the alternative pathway, ultimately leading to kidney injury. Factor B, which is exclusive to the alternative complement pathway, plays a distinct role in differentiating it from the other two pathways. It has been reported that Factor B can deposit along the GCW in a linear and granular pattern and exhibits good co-localization with C5b-9 (45). Compared to non-crescentic glomeruli, crescentic glomeruli exhibit significantly stronger deposition of Factor B (45). These findings provide clearer indications of alternative complement pathway activation. Notably, one major function of the alternative pathway is to amplify the activation of the classical pathway. Once the classical pathway is activated, components of the alternative pathway, such as Factor B, Factor D, and C3b, are recruited and participate in the complement activation process, interacting with the activation products of the classical pathway to further enhance complement activation (53, 54). Therefore, through this amplification loop, the alternative pathway not only engages in the complement activation process but also reinforces the activation effect of the classical pathway (56).

## 5 Comparing complement pathways in anti-GBM disease and other autoimmune disorders

Autoimmune diseases refer to a group of conditions in which the immune system abnormally turns against the body’s own healthy tissues and cells. In addition to anti-GBM disease, this category encompasses disorders such as systemic lupus erythematosus (SLE), rheumatoid arthritis, ANCA-associated vasculitis (AAV), and more (57). In comparison to anti-GBM disease, other autoimmune disorders also involve the activation of multiple complement pathways, but the antibodies forming immune complexes differ significantly. These include antinuclear antibodies, double-stranded DNA antibodies, ANCA, and rheumatoid factors, marking a fundamental distinction from anti-GBM disease (58, 59). In addition, in the context of anti-GBM disease, the activation of complement and the deposition of its components occur specifically on the glomerular basement membrane of the kidneys. This triggers a cascade of inflammatory reactions and cellular damage, ultimately resulting in the development of acute glomerulonephritis. However, in other autoimmune diseases, this pathological process occurs across multiple organs and tissues, such as the kidneys, skin, and joints, leading to a wide range of inflammatory responses and tissue injuries (60, 61). Notably, in SLE, complement levels reflect disease activity and severity to some extent, whereas measuring complement components in anti-GBM diseases has limited application in diagnostic and therapeutic monitoring (62, 63). In the management of these diseases, interventions targeting the complement pathway exhibit variations. In patients with anti-GBM disease, the primary approach involves the utilization of immunosuppressants and plasma exchange to suppress the production of self-antibodies and reduce complement activation (64, 65). Conversely, for other autoimmune diseases, treatment strategies may encompass the administration of immunosuppressants, nonsteroidal anti-inflammatory drugs (NSAIDs), and biologic agents to alleviate inflammatory responses and modulate the functionality of the immune system (66, 67). Excitingly, there is a growing anticipation that complement modulators hold promising potential as a potential therapeutic target for the majority of autoimmune diseases, including anti-GBM disease (57, 67, 68).

## 6 Double-seropositive for ANCA and anti-GBM antibodies

Among all autoimmune diseases, anti-GBM patients are most prone to develop anti-neutrophil cytoplasmic antibody (ANCA) positivity, typically in the form of anti-myeloperoxidase (MPO) antibodies. These individuals, referred to as antibody double-seropositive patients (DPPs), comprise approximately 20-35% of the total anti-GBM population (22). Conversely, up to 10% of ANCA-positive patients exhibit circulating anti-GBM antibodies as well (9, 69). In this patient population, a confluence of features is observed, combining ANCA-associated vasculitis and anti-GBM disease traits. These include severe renal illness, pulmonary hemorrhage, and diverse symptoms across various organs and tissues due to vascular damage (such as fever, fatigue, sinusitis, joint pain, etc.). Unfortunately, the majority of these patients demonstrate a suboptimal response to treatment and experience a poorer renal prognosis (70, 71). However, studies have also reported that DPPs have a higher average age compared to single-antibody-positive patients, and it is believed that the prognosis of DPPs depends on the titers of the antibodies (26, 72). Currently, known factors predicting survival rates include severe renal failure, advanced age, and the presence of pulmonary hemorrhage at the onset of the disease (73). Interestingly, ANCA positivity can also occur in circulating antibody-negative anti-GBM disease patients (74). Although the mechanisms and underlying connections between the simultaneous occurrence of these two diseases are not fully elucidated, research suggests that there are no significant specific differences in the antigens present in the serum between double-seropositive and single-antibody-positive patients (anti-GBM antibody or ANCA) (75). However, whether these two diseases involve interconnected complement activation pathways or mutually influence each other’s complement activation mechanisms remains uncertain.

Based on available data, anti-GBM antibodies can appear years to decades after ANCA antibodies. Both antibodies can coexist and persist for several years before experiencing a sharp increase in levels weeks to months before clinical disease onset (5). Traditionally, it has been believed that the self-degradation and remodeling of basement membranes lead to the exposure of hidden epitopes (76). However, recent research in AAV has uncovered that proteases released by ANCA-activated neutrophils digest Col (IV), leading to the exposure of α3(IV)NC1. CD11c+ macrophages then present GBM epitopes, triggering the host’s immune system to generate anti-GBM antibodies (12). In simple terms, AAV disrupts the hexameric structure of the GBM, exposing self-antigens stored within the GBM and triggering an anti-GBM response. Furthermore, due to varying affinities of antibodies for different tissues, both clinically and histologically, double-seropositive patients (DPPs) exhibit extrarenal manifestations similar to those observed in AAV patients, while their renal manifestations resemble those seen in anti-GBM disease patients (70). However, the precise role of the complement system in this process remains inadequately understood. Reports indicate a decrease in serum complement levels and an increase in renal complement deposition in patients with ANCA-associated glomerulonephritis (77, 78). Furthermore, in comparison to single antibody-positive patients, DPPs exhibit a lower trend in serum C3 and C4 levels, indicating a higher degree of complement activation in DPPs (79). And DPPs demonstrate a higher rate of primary disease recurrence, suggesting that the cascading complement activation might be one of the reasons for the more severe pathological changes observed in DPPs (73). It is worth noting that the total complement capacity appears to be limited within a short period, which may explain the less pronounced decrease in complement levels in DPPs compared to single antibody-positive diseases.

## 7 The impact of therapeutic plasma exchange on the complement system

Standard treatment for anti-GBM disease includes cyclophosphamide, corticosteroids, and therapeutic plasma exchange (TPE), a combined therapy that was first proposed in 1976 and continues to be used today (80). Currently, guidelines still recommend adopting the mentioned approach for treatment, even in the absence of pulmonary hemorrhage, except in cases of limited renal function (those on dialysis at presentation, with 100% crescents or > 50% global glomerulosclerosis in an adequate biopsy sample, and no pulmonary hemorrhage) (81, 82). Additionally, as long as renal function is viable, TPE should be continued until anti-GBM titers are no longer detectable (81). Some researchers have even proposed that high-dose intravenous glucocorticoids may not be necessary for anti-GBM disease following timely TPE and other necessary treatments (23). TPE involves the removal of plasma along with large molecular substances such as self-antibodies, immune complexes, and toxins through centrifugation or filtration. Studies have shown that the addition of TPE to corticosteroids and cyclophosphamide treatment improves overall survival, particularly in anti-GBM patients with initial treatment presenting a serum creatinine level higher than 6.8 mg/dL and concurrent pulmonary hemorrhage when conventional therapy fails to improve renal outcomes (83). DPPs exhibit multi-organ damage, including renal involvement, and TPE has been demonstrated to rapidly and effectively remove antibodies and inflammatory mediators, including complement (84). Immunoadsorption (IAS) is an alternative therapy to TPE, offering increased specificity. After blood separation in separator device, plasma passes through highly selective adsorption columns, targeting specific antibodies or complement molecules for precise and rapid removal of pathogenic factors. The impact of immunoadsorption on anti-GBM antibody titers is currently being investigated (NCT02765789). Existing research has demonstrated that IAS can effectively remove cellular cytokines, such as complement C3a and tumor necrosis factor-α. Moreover, IAS has shown comparable efficacy to TPE in clearing anti-GBM antibodies and promoting renal repair (85, 86).

In fact, most studies primarily focus on the clearance rate of antibodies during TPE, while the clearance effect on complement remains understudied. Furthermore, despite improvements, non-self materials can still activate complement during the process of blood passing through dialyzers and extracorporeal circuits, leading to dialysis-related inflammation (87, 88). Currently, complement C3 inhibitors (such as peptide-based matrix metalloproteinase inhibitor 101 or complement c5 convertase inhibitor 40) may hold promise in intervening in dialysis-related inflammation for patients undergoing chronic dialysis (89, 90). Additionally, the risks associated with dialysis have also spurred the development of complement inhibitors for non-dialysis patients.

## 8 The application of complement regulators in anti-GBM disease

There is no doubt that significant progress has been made in the development of drugs targeting the complement system. Serine proteases, including Cinryze, Cetor, Berinert, Ruconest, and C5 inhibitors such as eculizumab, have been approved for the treatment of complement-related disorders (2, 91). Eculizumab, an anti-C5 humanized monoclonal antibody, can block the cleavage of C5 and inhibit the formation of C5b-9 (92). Currently, it is primarily approved for paroxysmal nocturnal hemoglobinuria and atypical hemolytic uremic syndrome, or used for its non-specific anti-inflammatory effects in the intervention of AAV (93). Considering the crucial role of C5 in complement activation, targeting C5 may represent an effective therapeutic approach for anti-GBM disease, although C5 blockade does not reverse the initial driving factors of the disease. High circulating levels of C5b-9, along with positive staining in renal biopsies of anti-GBM patients, confirm the activation of the terminal complement cascade, providing a rationale for the use of Eculizumab. It is important to note that the C5-blocking effect of Eculizumab can alleviate glomerular inflammation and reduce proteinuria, but it does not decrease complement deposition in the kidneys (94). In life-threatening organ damage seen in Goodpasture syndrome, Eculizumab even has the potential to block complement-driven lung injury (95). Therefore, despite the high cost of treatment, C5 and C5a inhibitors may serve as targeted anti-inflammatory agents to mitigate renal injury.

In addition to treating primary anti-GBM disease, further research is warranted on targeting the complement system to improve outcomes related to post-transplant recurrence. Among 224 patients who underwent renal allograft transplantation for anti-GBM disease between 1963 and 2010, six patients experienced a recurrence of anti-GBM disease, with graft failure occurring in two cases (96). As early as 20 years ago, when immunosuppressive agents were limited, studies reported a high recurrence rate of 14% in anti-GBM disease (97). Therefore, considering the higher incidence of disease recurrence following allograft kidney transplantation in the presence of anti-GBM antibodies, it is crucial to ensure low titers of anti-GBM antibodies before transplantation. Indeed, despite delaying transplantation and implementing standardized immunosuppressive regimens, sporadic cases of recurrence still occur (98, 99). Furthermore, disease recurrence has been observed even in the absence of serum anti-GBM antibodies (100, 101). The occurrence of such cases may be attributed to the presence of specific subtypes of anti-GBM antibodies that are not detectable by the tests currently employed (102, 103). Additionally, it cannot be ruled out that the levels of anti-GBM antibodies may fall below the detection range, while the amplifying effect of the complement cascade could potentially contribute to disease recurrence. Currently, research has shown that blocking complement activation through the use of serine protease inhibitors, such as C1 esterase inhibitors, or terminal complement pathway inhibitors can prevent delayed graft function and reduce rejection reactions, ultimately improving graft survival (104, 105). Currently, the use of eculizumab is being investigated in kidney transplant recipients to counteract antibody-mediated rejection and complement-mediated injury (NCT02113891, NCT01327573).

## 9 Considerations for complement-targeted therapy

The deficiency of complement components can also lead to the accumulation of immune complexes and cellular debris, reflecting the intricate role of complement in renal diseases (106). For instance, in an experimental animal model of anti-GBM disease, the knockout of C3 worsened glomerular injury (28). This may be attributed to the loss of C3-mediated clearance of antigen-antibody complexes, resulting in increased deposition of immune complexes and exacerbation of glomerular injury. Furthermore, studies have reported that defects in factor H of the alternative pathway lead to uncontrolled complement activation and deposition of complement on glomeruli, while supplementation of factor H can inhibit C3 deposition on the GBM (106, 107). Therefore, not all detected complement components are pathogenic, and the activation of the complement system is not always harmful to the body. When using complement-targeted therapies, it is essential to rigorously evaluate the effects on complement to avoid the inappropriate use of complement modulators. Furthermore, inhibiting or modulating the function of the complement system may reduce the body’s ability to resist infections, making patients more susceptible to pathogen invasion. Studies have reported that patients treated with eculizumab may face a significant risk of severe infections caused by microorganisms such as gonococcus and meningococcus (108, 109). Therefore, during complement-targeted treatment, it is crucial to closely monitor the risk of treatment-related infections and assess the potential benefits and risks. The continuation of this treatment approach is only meaningful when the benefits outweigh the risks.

Notably, complement inhibition therapy is an emerging treatment strategy, and currently, there are no available biomarkers to predict the response of renal diseases to complement inhibition (110). Thus, the identification of biomarkers that can predict the response of kidney disease patients to complement blockade therapy is one of the current research priorities. Due to the complexity of renal diseases and individual variations, the response of patients to complement inhibition therapy can vary significantly. The use of complement inhibitors requires individualized strategies based on the severity of the condition and complement levels. For instance, during the acute phase of complement-mediated renal diseases, the choice of complement modulators tends to focus on directly targeting key steps in complement activation, such as C3/C5 antibodies or C3a/C5a receptor antagonists. These drugs can effectively inhibit the effects of C3a/C5a and the formation of MAC, thereby reducing the release of inflammatory mediators and cell damage (110). Conversely, in the chronic phase, the treatment goal is to alleviate complement-mediated inflammation and fibrosis while preserving renal function stability. For example, the therapeutic effects of small interfering RNA may be delayed but could prove cost-effective in long-term strategies (111). Overall, in different scenarios, the selection of appropriate complement modulators may require a more comprehensive consideration of the underlying pathological mechanisms of the disease and individual characteristics.

## 10 Conclusion

In conclusion, the role of complement in anti-GBM disease is both complex and crucial. It plays a dual role in regulating the antigen-antibody immune response and activating the inflammatory process through complement cascade reactions, thereby directly or indirectly causing damage to the glomeruli. It is evident that all three complement activation pathways are involved in the pathogenesis of anti-GBM disease. Moreover, complement activation is present not only in patients with classic anti-GBM disease, but also in patients who are double positive for ANCA and anti-GBM antibodies. The degree of complement activation is positively correlated with the severity of the disease. Research on the complement system contributes to a deeper understanding of the pathogenesis of anti-GBM disease and provides a foundation for the development of complement-targeted therapeutics.

## Author contributions

This study was a collaborative effort among all authors. AT and XZ were involved in writing the manuscript. TT, DX, and BX contributed to manuscript revisions. YH, and ML provided final approval. All authors contributed to the article and approved the submitted version.
